# Efficacy of Fosfomycin against Planktonic and Biofilm-Associated MDR Uropathogenic *Escherichia coli* Clinical Isolates

**DOI:** 10.3390/tropicalmed7090235

**Published:** 2022-09-08

**Authors:** Haziel Eleazar Dzib-Baak, Andrés Humberto Uc-Cachón, Angel de Jesús Dzul-Beh, Rey Fernando Rosado-Manzano, Carlos Gracida-Osorno, Gloria María Molina-Salinas

**Affiliations:** 1 Unidad de Investigación Médica Yucatán, Instituto Mexicano del Seguro Social, Mérida 97150, Mexico; 2 Unidad Médica de Alta Especialidad, Instituto Mexicano del Seguro Social, Mérida 97150, Mexico; 3 Hospital General Regional No. 1, Instituto Mexicano del Seguro Social, Mérida 97150, Mexico

**Keywords:** uropathogenic *Escherichia coli*, fosfomycin, biofilm, multidrug-resistant

## Abstract

Urinary tract infections (UTI) are a severe public health problem and are caused mainly by the uropathogenic *Escherichia coli* (UPEC). Antimicrobial resistance and limited development of new antimicrobials have led to the reuse of old antibiotics such as fosfomycin. The aim of this study was to evaluate the in vitro efficacy of fosfomycin on a collection of multidrug-resistant (MDR) UPEC and the degradative activity on biofilm producers. A total of 100 MDR UPEC clinical isolates were collected from patients at Mexican second- and third-level hospitals. Microorganism identification was performed using an automated system, the evaluation of the susceptibility of clinical isolates to fosfomycin was performed using the resazurin microtiter assay, and the identification of biofilm producers and the effect of fosfomycin in biofilms were evaluated using the crystal violet method. Among planktonic MDR UPEC, 93% were susceptible to fosfomycin. Eighty-three MDR UPEC were categorized as weak (39.8%), moderate (45.2%), and strong (14.5%) biofilm producers. Fosfomycin exhibited degradative activity ranging from 164.4 µg/mL to 1045 µg/mL. Weak producers required statistically lower concentrations of fosfomycin to destroy the biofilm, contrary to moderate and strong producers. In conclusion, fosfomycin could be an option for the treatment of infections caused by MDR UPEC, for which the antimicrobial treatment is more often becoming limited.

## 1. Introduction

Urinary tract infection (UTI) is one of the most common infectious diseases, and the majority of these illnesses are caused by uropathogenic *Escherichia coli* (UPEC) [1]. UPEC accounts for up to 90% of community-acquired UTI and for approximately 50% of nosocomial UTI [2]. The emergence of antimicrobial resistance (AMR) in bacteria has become a global public health threat because it compromises the success of therapies, increasing costs and hospital stays. Bacterial resistance is the ability of bacteria to evade the action of antimicrobial drugs. The epidemiological view is intensified by the emergence of multidrug-resistant (MDR) bacteria, which are defined as those showing resistance to at least one drug from among three or more chemical groups of antimicrobials, and extensively drug-resistant (XDR) bacteria that are only susceptible to the two remaining groups of antimicrobial agents. These phenotypes are closely related with higher mortality rates [3,4].

MDR UPEC clinical isolates can evade the effects of antibiotics via a variety of mechanisms, and one of those most associated with UTI is the formation of biofilms. A biofilm is defined as a structured community of bacterial cells embedded in a self-produced polymeric matrix that is attached to an inert or living surface [5,6,7]. It has been documented that UPEC strains can form biofilms attached to genitourinary-tract tissue. This characteristic may avoid bacterial eradication during antibiotic treatment [8].

AMR and the scarce development of new antibacterial drugs has led to the reuse or repurposing of antibiotics whose use has been discontinued. In this era of increasing AMR, it is necessary to discover novel drugs that are active on biofilms [9,10].

Fosfomycin is an old antibiotic first isolated in 1969 from cultures of *Streptomyces* spp.; it represents a class of antibiotics that acts by inhibiting the early stages of bacterial cell-wall biosynthesis in an irreversible manner with a bactericidal effect. The spectrum of fosfomycin activity is broad and covers most Gram-positive and Gram-negative bacteria, including the Enterobacteriaceae family, of which *Escherichia coli* is a member [11].

The use of fosfomycin was displaced by the appearance of new-generation drugs and the association with adverse events. Currently, due to improved pharmacokinetics, new dosage adjustments and new formulations with greater bioavailability, such as fosfomycin–tromethamine, have improved the safety of its use [12,13]. Due to the high incidence of MDR bacteria, its use has increased dramatically in recent years, since fosfomycin, alone or in combination, constitutes a therapeutic alternative. Fosfomycin was shown to be highly active against MDR UPEC clinical isolates in previous studies [14,15].

Fosfomycin was recently shown to have in vitro activity on biofilms formed by UPEC. This feature is important, because the role of biofilm formation during the pathogenesis of UTI has been extensively studied; the production of this extracellular matrix aids adhesion, provides protection against immune response, and favors bacterial persistence and chronicity [16]. In addition, some studies have shown that fosfomycin has activity toward biofilm-associated MDR UPEC strains, although these studies are still scarce [17,18].

In our region, the clinical use of fosfomycin is limited, and in vitro susceptibility studies against bacterial clinical isolates are scarce. Furthermore, there is a high prevalence of MDR bacterial clinical isolates [19]; thus, these antibiotics could comprise an option for treating infections caused by these MDR pathogens. Therefore, the aim of this study was to determine the in vitro efficacy of fosfomycin against planktonic and biofilm-associated MDR UPEC clinical isolates.

## 2. Materials and Methods

This project was approved by the Scientific and Ethics Committees National of the Instituto Mexicano del Seguro Social (IMSS), with approval number R-785-2019-073.

### 2.1. Bacterial Strains

A total of 100 nonduplicated MDR UPEC clinical isolates were obtained from hospitalized and ambulatory patients admitted to second- and third-level hospitals of Instituto Mexicano del Seguro Social, (Hospital General Regional No. 1 and Unidad Médica de Alta Especialidad) in Mérida, Mexico. Microorganism identification was performed using the automated MICROSCAN WalkAway^®^ system (Beckman Coulter, Inc., Brea, CA, USA). The MICROSCAN panels used modified chromogenic and conventional tests for the identification of *E. coli*.

A total of 15 different categories of antibiotics according the Magiorakos et al. (2012) [4] categorization were tested against MDR UPEC strains, as presented in Table 1.

All clinical isolates of MDR UPEC were maintained at −80 °C in tryptic soy broth (TSB, Difco, Becton, Dickinson, and Co. NJ, USA), supplemented with 20% *v/v* glycerol (Sigma-Aldrich, St. Louis, MO, USA).

### 2.2. Antimicrobial Agents

Fosfomycin disodium (Sigma-Aldrich) was used. All solutions were sterilized by filtration through a 0.22 μm nylon acrodisc (MilliporeSigma, MO, USA), and then stored at −80 °C until their use.

### 2.3. Fosfomycin Effect on Planktonic MDR UPEC

To evaluate the susceptibility of planktonic MDR UPEC strains to fosfomycin, the minimal inhibitory concentration (MIC) was determined using the Resazurin Microtiter Assay (REMA) broth microdilution method utilizing 96-well microplates, as described by Sarker et al. (2007), with some modifications [20].

First, the clinical isolates were cultured on Muller–Hinton Agar (MHA; Becton, Dickinson, and Co.) plates at 37 °C for 24 h. After 24 h, the bacterial colonies were suspended in Muller–Hinton Broth (MHB; Becton, Dickinson, and Co.) and grown at 37 °C to match the turbidity of the 0.5 McFarland determined with a densitometer (Den-1; Biosan, Riga, Lat-via). This culture was further diluted at 1:50 and 100 μL of the bacterial suspension (10^6^ colony-forming units (CFU)/mL) and incubated and cultured with 100 μL of MHB containing fosfomycin at serial dilutions (512–16 μg/mL). For testing the dilution of fosfomycin, the MHB was supplemented with 25 µg/mL glucose-6-phosphate (G-6-P; Sigma-Aldrich) as a transporter of antibiotic. Then, 100 μL of the bacterial suspension was added to all the microwells and incubated at 37 °C for 16 h. Growth controls (without drug) and sterility (only MHB) were included. After incubation, 30 μL of the 0.015% (*w*/*v*) of the resazurin (Sigma-Aldrich) solution was added and incubated again at 37 °C for 2 h. The MIC was determined as the lowest concentration of the antimicrobial agent inhibiting bacterial growth, which is defined as that which prevents the color change of resazurin from blue to any pink hue. The MIC values of fosfomycin were analyzed according to the guidelines of the Clinical and Laboratory Standards Institute (CLSI) [21]. Each assay was performed one independent time in duplicate.

### 2.4. Biofilm-Formation Assay for MDR UPEC

To identify which of the 100 clinical isolates produce biofilm, the crystal violet staining method was utilized (CV; Hycel, Jalisco, Mexico) in 96-well microplates, as described by Christensen, with some modifications [19,22].

Briefly, the clinical isolates were cultured on MHA plates at 37 °C for 24 h. Then, the clinical isolates were inoculated in TSB and incubated at 37 °C for 24 h. The bacterial culture was transferred into fresh TSB and grown to match the turbidity of 1.0 McFarland with a densitometer. This bacterial suspension was further diluted at 1:100 in TSB supplemented with 1% (*w*/*v*) dextrose (Wöhler, Mexico City, Mexico), and 200 μL of the suspension was added to each well of microplate and incubated at 37 °C for 24 h. Following this, the bacterial culture broth was decanted, and the wells were washed three times with sterile distilled water and dried at 60 °C for 45 min.

The biofilm was stained with 200 μL of a 0.1% (*w*/*v*) CV solution followed by development for 30 min at room temperature. The excess CV was removed by washing three times with sterile distilled water, rather than adding 200 μL of a 30% (*v*/*v*) acetic-acid (Fermont, Nuevo León, México) solution to release the dye from the biofilm. Wells without bacterial suspension were included as a negative control. The optical density (OD) of stained adherent bacteria was determined using a microplate reader (IMark; Bio-Rad, CA, USA) at a wavelength of 490 nm. These OD_490_ values were considered as an index of bacteria adhering to the surface and forming biofilms. Each assay was performed in triplicate, the data were averaged, and the standard deviation (SD) was calculated. The capacity of biofilm producers was defined with the criteria of Akbari et al. (2017) [23], as presented in Table 2.

### 2.5. Fosfomycin Effect on Biofilm-Associated MDR UPEC

The biofilm degradation assay was performed using the method described by Gopichand (2019), with some modifications [14]. To evaluate the effect of fosfomycin on the biofilms of MDR UPEC producer strains, the assay was added into 96-well plates, as previously described.

After biofilm production, the culture medium was decanted, and the wells were washed twice with sterile water to remove the planktonic bacteria. Then, 200 µL of fresh TSB broth with fosfomycin at concentrations of 1024–64 μg/mL was added into the wells. Wells with ethylenediaminetetraacetic acid (EDTA; Sigma Aldrich) and without fosfomycin solutions were utilized as positive and negative controls, respectively. The microplate was incubated at 37 °C for 24 h; subsequently, the content of the well was removed, washed, and stained as previously described. The OD_490_ of the dye CV stain was determined in all wells. Following this, the concentration of the fosfomycin that degraded 50% (DC_50_) of the biofilm was calculated using GraphPad Prism version 8 software (GraphPad Software Inc., San Diego, CA, USA). The assays were performed in triplicate.

### 2.6. Statistical Analysis

An analysis of variance test (ANOVA) and the post hoc Tukey method for multiple comparisons were performed to analyze whether there were significant differences in the effect of fosfomycin in relation to capacity of the isolates to produce biofilms. The correlation of biofilm production among resistant and susceptible isolates was calculated using the Fisher exact test with GraphPad Prism version 8 statistical software (GraphPad Software Inc.). A *p*-value ≤ 0.05 was considered statistically significant.

## 3. Results

### 3.1. Susceptibility of Planktonic MDR UPEC to Fosfomycin

Clinical isolates of MDR UPEC that exhibited a high rate of susceptibility to fosfomycin demonstrated an efficacy of 93% against free floating bacteria.

Taking into account all of our results, the activity of 16 chemical groups of antibiotics was evaluated on the collection of MDR UPEC according to Magiorakos et al. (2012) [4]. The MDR UPEC revealed resistance rates of up to six or even 13 chemical groups of antibiotics (Table S1). The clinical isolate UPEC-120 was resistant to the drugs included in 13 chemical groups, and three of these, UPEC-76, -77, and -126, were resistant to drugs included in 12 chemical groups; these drug resistance and susceptibility profiles are depicted in Table 2. It should be noted that the clinical isolates UPEC-76 and UPEC-77 showed resistance to fosfomycin (Table 3).

### 3.2. Biofilm Producer Capacity of MDR UPEC

**Figure 1:**
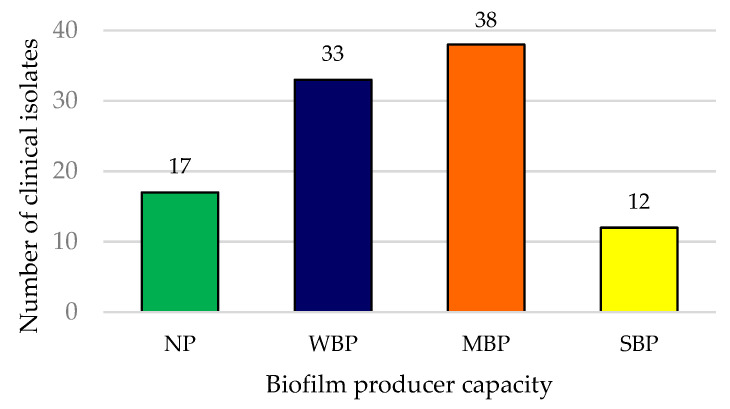
Distribution of UPEC clinical isolates by biofilm-formation capacity. NP: not a biofilm producer; WBP: weak biofilm producer; MBP: moderate biofilm producer; SBP: strong biofilm producer.

A total of 83 UPEC clinical isolates were biofilm producers. The majority of these were moderate (45.2%), followed by weak (39.8%), and strong (14.5%) biofilm producers (Figure 1).

The differences in the AMR rates of some MDR UPEC allowed for the analysis of the relationships between their ability to produce biofilm and their AMR; however, among these differences, there was no significant relationship (Table 4).

### 3.3. Effect of Fosfomycin on Biofilm-Associated MDR UPEC

The effect of fosfomycin on biofilm degradation was evaluated on 83 MDR UPEC clinical isolates, and the results are shown in Table 5.

Fosfomycin exhibited degradative activity ranging from 164.4 µg/mL to 1045 µg/mL and revealed weak activity on biofilms from the moderate biofilm producer UPEC-48 (DC_50_ = 1045 µg/mL) and from the strong producer UPEC-121 (DC_50_ = 1021.2 µg/mL) (Table 5).

**Figure 2:**
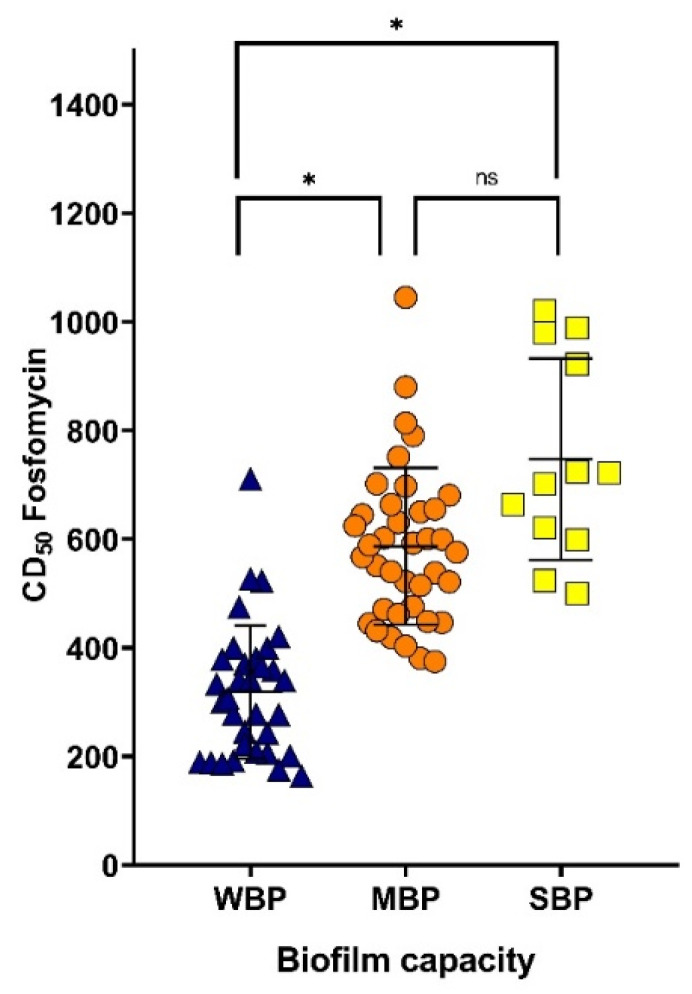
Distribution of the DC_50_ effect of fosfomycin in relation to the biofilm capacity of the UPEC isolates. One-way ANOVA was performed, and the Tukey post hoc test was conducted to compare the concentration of fosfomycin that degraded 50% of the biofilms in the different groups of biofilm producers, where significant differences are indicated as follows: ns, not significant (*p* > 0.05); * p < 0.0001. WBP: weak biofilm producer; MBP: moderate biofilm producer; SBP: strong biofilm producer.

The distribution of the values of DC_50_ of fosfomycin on the biofilm of UPEC clinical isolates in relation with the biofilm-formation capacity is shown in Figure 2. In general, weak producers of biofilm required lower concentrations of fosfomycin to destroy the biofilm (DC_50_ = 164.4–523.1 µg/mL), contrary to moderate (DC_50_ = 403.1–751.4 µg/mL) and strong (DC_50_ = 523.9–980.1 µg/mL) producers. The difference in the variances of the DC_50_ values of fosfomycin against biofilms among the three categories of biofilm producers revealed that these were statistically significant.

## 4. Discussion

Increasing bacterial resistance to antibiotics in recent years is a serious health problem that has reduced treatment options for infections, including UTIs. These infections are recognized as among the most common infectious diseases in the world, and UPEC strains are the most prevalent causative agents of UTI [24]. Currently, this has led to the reuse or repurposing of antibiotics whose use has been discontinued, such as fosfomycin. The present study determined the in vitro susceptibility of fosfomycin toward planktonic and biofilm-associated MDR UPEC.

Our results showed that fosfomycin possesses high in-vitro efficacy on planktonic MDR UPEC clinical isolates. Some authors reported the activity of fosfomycin on the clinical isolates of non-MDR and MDR UPEC. The susceptibility of clinical isolates of UPEC to fosfomycin was reported in hospitals from Germany, Morocco, India, and China, with susceptibility between 91% and 98% [25,26,27,28]. In Mexico, Ballesteros-Monreal et al. (2020) reported that the susceptibility rate of UPEC clinical isolates to fosfomycin from the patients of two states, Sonora and Puebla, was 96% [29]. Similarly, Lagunas-Rangel et al. (2018) reported that 90% of UPEC isolates from patients at a private sanatorium in Morelia, Michoacán, Mexico, were susceptible to Fosfomycin [30]. In addition to the high in vitro efficacy of Fosfomycin, it has a unique mechanism of action among all classes of antimicrobials that prevents cross-resistance with other antimicrobials [31].

Antibiotics of 16 different chemical groups of antibiotics were tested on our MDR UPEC collection. Our MDR UPEC set demonstrated resistance rates to drugs, including six or even 13 chemical groups of antibiotics. According to Magiorakos et al. (2012), antimicrobials to treat *E. coli* infections can be grouped in 17 chemical groups; although the arsenal to cure *E. coli* infections is very broad, our results revealed MDR UPEC clinical isolates resistant to drugs of 12 or even of 13 chemical groups of antibiotics; these bacteria are close to being XDR (non-susceptible to ≥1 agent in 17 chemical groups) [4].

The identification of XDR UPEC is scarcely reported, in contrast to that of other uropathogenic bacterial species such as *Pseudomonas aeruginosa* or *Acinetobacter baumannii* [32,33]. Infection by an XDR bacterium is closely associated with prolonged hospital stays and higher rates of mortality due to limited options for antimicrobial therapy. Early identification and close monitoring of XDR bacterial strains are essential to reduce the threat of AMR, which is now a global problem; virulent XDR bacterial strains could kill millions of persons in the not-so-distant future [34,35].

It has been estimated that up to 65% of human infections are caused by bacteria that have the capacity to produce biofilms. It is also known that biofilms protect germs from host defense mechanisms and the action of antimicrobials [36]. Uropathogenic bacteria have been documented to form biofilms attached to urogenital tissue. These biofilms create an inactive reservoir, which can persist undetected and switch to a planktonic form, occasionally causing recurrent UTI [37]. In this study, 84% of MDR UPEC clinical isolates exhibited in vitro biofilm-formation ability. Studies in other countries have registered UPEC clinical isolates with biofilm-production ability, such as Iran (99%) [38], Pakistan (100%) [39], Nepal (75%) [40], Thailand (54%) [41], and India (47%) [42]. In our study, the majority of the MDR UPEC clinical isolates were moderate (39%) and weak (33%) biofilm producers, while 73% of UPEC clinical isolates from patients in Pakistan were strong producers, unlike 72% of UPEC from patients in Iran, with weak producers of biofilms. These differences might be due to the genetic diversity of UPEC strains and could be affected by the methodology for culturing or the culture media for distinguishing the biofilms production ability [38,43].

Some authors analyzed the possible relationship between AMR and biofilm production among clinical isolates; however, as also shown in our study, these authors did not find a significant association between biofilm production and biofilm resistance to antibiotics in MDR UPEC [16,44].

The biofilm-formation ability of UPEC enables them to remain in the urinary tract for a longer period. This can play a role in increasing the severity of the UTI, its recurrence, and its difficulty to treat. An alternative to degrade these biofilms is necessary. In our study, we analyzed the effect of fosfomycin in biofilm degradation activity ranging from 164.4 µg/mL to 1045 µg/mL. There were statistically significant differences in biofilm producers and in the concentrations of fosfomycin needed to degrade their biofilm, showing that a higher amount of biofilm biomass required a higher amount of fosfomycin to degrade it. Fosfomycin is not metabolized but is instead excreted unchanged in the urine via glomerular filtration. An oral administration of a single, 3 g fosfomycin dose (dose usually used in adults) achieves peak urinary concentrations reaching ∼4000 µg/mL [45], higher than that necessary to degrade the MDR UPEC biofilms in this study.

Other studies evaluated the in vitro effect of fosfomycin on UPEC biofilms. Fosfomycin at concentrations of 300, 700, and 1500 µg/mL significantly reduced the production of MDR UPEC biofilms [46]. In addition, the effect of fosfomycin in combination with different antibiotics, such as amikacin or ciprofloxacin, demonstrated high efficacy (70–90%) in terms of the biofilm inhibition of MDR UPEC [17]. While these previous studies revealed the effect of fosfomycin on the inhibition of biofilm-formation [17,18,46], our study is, to our knowledge, the first report of the in vitro effect of fosfomycin alone on the degradation of MDR UPEC biofilms.

## 5. Conclusions

In conclusion, our data suggest that fosfomycin might be an alternative for the treatment of infections caused by MDR UPEC. Our investigation showed that our MDR UPEC clinical isolates are close to being XDR, and epidemiological surveillance of these strains is important, in that therapeutic options are becoming more limited. The majority of our MDR UPEC clinical isolates possess biofilm-production ability, and fosfomycin represents an antibiotic option which exhibited efficacy in degrading the biofilm produced.

## Figures and Tables

**Table 1:** Antibiotics tested against MDR UPEC.

Antimicrobial Category	Antibiotic
Penicillins	AMP, PIP
Penicillins + beta-lactamase inhibitors	SAM
Antipseudomonal penicillins + beta-lactamase inhibitors	TZP
1st- and 2nd-generation cephalosporins	CFX
3rd- and 4th-generation cephalosporins	FEP, CTX, CRO, CAZ
Cephamycins	CTT
Aminoglycosides	AMK, GEN, TOB
Carbapenems	IMP, MEM, ETP
Fluoroquinolones	LVX, CIP
Folate pathway inhibitors	SXT
Monobactams	ATM
Glycylcyclines	TGG
Tetracyclines	TET
Phenicols	CLO
Polymyxins	CST

AMP: ampicillin; PIP: piperacicllin; SAM: ampicillin/sulbactam; TZP: piperacillin/tazobactam; FEP: cefepime; CTX: cetofaxime; CRO: ceftriaxone, CAZ: ceftazidime; CFX: cefuroxime; CTT: cefotetan; AMK: amikacin; GEN: gentamicin; TOB: tobramycin; MEM: meropenem; IPM: imipenem; ETP: ertapenem; LVX: levofloxacin; CIP: ciprofloxacin; SXT: trimethoprim/sulfamethoxazole; ATM: aztreonam; TGG: tigecycline; TET: tetracycline; CLO: chloramphenicol; CST: colistin.

**Table 2:** Criteria used for the classification of UPEC according to biofilm capacity.

Criteria	Biofilm-Formation Capacity
ODs ≤ODc	Not a biofilm producer
ODc ≤ ODs ≤ 2 × ODc	Weak biofilm producer
2 × ODc ≤ ODs ≤ 4 × ODc	Moderate biofilm producer
4 × ODc < ODs	Strong biofilm producer

ODs (average of the optical density [OD] of sample); ODc (average OD of negative control + 3 × SD).

**Table 3:** Resistance and susceptibility profile of MDR UPEC clinical isolates with highest resistance rates.

ID Isolate	Resistance Drugs	Susceptibility Drugs
120	AMP, PIP, SAM, TZP, FEP, CTX, CFX, CRO, CAZ, CTT, AMK, GEN, TOB, IMP, ERT, LVX, CIP, SXT, ATM, TET, TGG	CLO, FOF, CST
76	AMP, SAM, PIP, FEP, CTX, CFX, CRO, CAZ, GEN, TOB, LVX, CIP, SXT, ATM, TET, CLO, FOF, CST	TZP, CTT, AMK, IMP, MEM, ETP, TGG
77	AMP, SAM, PIP, TZP, FEP, CTX, CFX, CRO, CAZ, TOB, IMP, LVX, CIP, SXT, ATM, TET, CLO, FOF	CTT, GEN, IMP, MEM, ETP, TGG, CST
126	AMP, SAM, PIP, TZP, FEP, CTX, CFX, CRO, CAZ, AMK, TOB, LVX, CIP, SXT, ATM, TET, CLO	CTT, AMK, GEN, IMP, MEM, ETP, TGG, CST, FOF, CST

AMP: ampicillin; PIP: piperacicllin; SAM: ampicillin/sulbactam; TZP: piperacillin/tazobactam; FEP: cefepime; CTX: cetofaxime; CRO: ceftriaxone; CAZ: ceftazidime; CFX: cefuroxime; CTT: cefotetan; AMK: amikacin; GEN: gentamicin; TOB: tobramycin; MEM: meropenem; IPM: imipenem; ETP: ertapenem; LVX: levofloxacin; CIP: ciprofloxacin; SXT: trimethoprim/sulfamethoxazole; ATM: aztreonam; TGG: tigecycline; TET: tetracycline; CLO: chloramphenicol; FOF: fosfomycin; CST: colistin.

**Table 4:** Relationship between biofilm production and AMR.

Antimicrobials		Biofilm Producers	Non-Producers	*p*-Value
Amikacin	R	18	2	0.51
	S	66	14	
Piperacillin/tazobactam	R	37	6	0.78
	S	47	10	
Gentamicin	R	40	7	>0.99
	S	44	9	
Tobramycin	R	55	11	>0.99
	S	29	5	
Trimethoprim/sulfamethoxazole	R	54	12	0.57
	S	30	4	
Tetracycline	R	66	12	0.75
	S	18	4	

R: resistant to antibiotic; S: susceptible to antibiotic.

**Table 5:** Fosfomycin efficacy (DC_50_) on biofilm-associated MDR UPEC clinical isolates.

ID Isolate	Biofilm-Formation Capacity	Anti-Biofilm Activity DC_50_ (μg/mL)	ID isolate	Biofilm-Formation Capacity	Anti-Biofilm Activity DC_50_ (μg/mL)
61	WBP	164.4 ± 1.9	140	MBP	514.7 ± 3.0
117	WBP	175.6 ± 2.2	93	MBP	521.8 ± 6.9
116	WBP	187.1 ± 1.8	7	MBP	522.0 ± 3.1
95	WBP	188.6 ± 4.7	1	WBP	523.1 ± 1.8
43	WBP	189.6 ± 0.7	70	SBP	523.9 ± 5.9
48	WBP	192.0 ± 2.5	127	WBP	526.8 ± 5.7
38	WBP	200.8 ± 1.0	67	MBP	538.4 ± 3.1
27	WBP	206.0 ± 5.1	56	MBP	540.0 ± 4.8
119	WBP	209.0 ± 1.4	21	MBP	551.0 ± 0.9
65	WBP	222.4 ± 4.1	40	MBP	567.7 ± 2.7
52	WBP	243.1 ± 4.6	105	MBP	576.2 ± 2.3
16	WBP	245.1 ± 1.9	63	MBP	589.1 ± 5.7
54	WBP	276.9 ± 1.4	49	MBP	593.2 ± 1.2
34	WBP	278.0 ± 3.0	138	SBP	599.5 ± 4.8
91	WBP	278.0 ± 5.9	87	MBP	599.8 ± 1.4
15	WBP	301.1 ± 3.7	55	MBP	601.2 ± 1.8
122	WBP	307.5 ± 3.3	20	MBP	602.2 ± 2.1
39	WBP	333.2 ± 1.4	112	SBP	620.9 ± 8.5
68	WBP	339.8 ± 1.2	81	MBP	624.6 ± 2.9
53	WBP	340.0 ± 1.6	109	MBP	631.0 ± 2.5
88	WBP	341.8 ± 1.2	74	MBP	645.1 ± 2.1
2	WBP	359.0 ± 2.1	62	MBP	650.6 ± 1.3
33	WBP	364.7 ± 1.0	124	MBP	655.7 ± 4.7
11	WBP	369.6 ± 1.7	71	SBP	663.9 ± 2.6
118	MBP	375.2 ± 5.7	129	MBP	664.0 ± 1.6
92	WBP	378.2 ± 5.2	36	MBP	680.9 ± 2.6
41	MBP	380.8 ± 3.2	60	MBP	698.0 ± 2.3
18	WBP	380.9 ± 2.8	136	SBP	701.5 ± 2.7
5	WBP	400.0 ± 6.1	22	MBP	702.1 ± 1.4
19	WBP	400.1 ± 2.4	29	WBP	710.9 ± 2.6
66	MBP	403.1 ± 1.9	64	SBP	722.0 ± 1.1
10	MBP	419.0 ± 1.2	3	SBP	723.6 ± 2.0
26	WBP	420.7 ± 0.9	80	MBP	751.4 ± 1.8
47	MBP	431.6 ± 1.4	120	MBP	790.8 ± 4.1
28	MBP	444.8 ± 1.3	142	MBP	814.2 ± 5.1
50	MBP	447.0 ± 1.7	128	MBP	880.4 ± 1.7
114	MBP	449.1 ± 1.6	108	SBP	922.6 ± 6.3
139	MBP	461.5 ± 1.2	77	SBP	980.1 ± 1.1
97	MBP	470.9 ± 3.1	126	SBP	989.2 ± 2.6
131	WBP	474.8 ± 1.3	121	SBP	1021.2 ± 1.3
107	MBP	476.3 ± 5.7	76	MBP	1045.0 ± 3.4
101	SBP	500.2 ± 7.8			

WBP: weak biofilm producer; MBP: moderate biofilm producer; SBP: strong biofilm producer.

## Data Availability

Data is contained within the article or supplementary material.

## Supplementary Materials

The following supporting information can be downloaded at https://www.mdpi.com/article/10.3390/tropicalmed7090235/s1: Table S1. Resistance profile of MDR UPEC.

## References

[1] Flores-Mireles A.L., Walker J.N., Caparon M., Hultgren S.J. (2015). Urinary tract infections: Epidemiology, mechanisms of infection and treatment options. Nat. Rev. Microbiol..

[2] Asadi-Karam M.R., Habibi M., Bouzari S. (2019). Urinary tract infection: Pathogenicity, antibiotic resistance, and development of effective vaccines against uropathogenic *Escherichia coli*. Mol. Immunol..

[3] Spellberg B., Blaser M., Guidos R.J., Boucher H.W., Bradley J.S., Eisenstein B.I., Gerding D., Lynfield R., Reller L.B., Rex J. (2011). Combat antimicrobial resistance: Policy recommendations to save lives. Clin. Infect. Dis..

[4] Magiorakos A.P., Srinivasan A., Carey R.B., Carmeli Y., Falagas M.E., Giske C.G., Harbarth S., Hindler J.F., Kahlmeter G., Olsson-Liljequist B. (2012). Multidrug-resistant, extensively drug-resistant and pandrug-resistant bacteria: An international expert proposal for interim standard definitions for acquired resistance. Clin. Microbiol. Infect..

[5] Roy R., Tiwari M., Donelli G., Tiwari V. (2018). Strategies for combating bacterial biofilms: A focus on anti-biofilm agents and their mechanisms of action. Virulence.

[6] Vestby L.K., Torstein G., Simm R., Nesse L.L. (2020). Bacterial biofilm and its role in the pathogenesis of disease. Antibiotics.

[7] Marti R., Schmid M., Kulli S., Schneeberger K., Naskova J., Knøchel S., Hummerjohann J. (2017). Biofilm formation potential of heat-resistant *Escherichia coli* dairy isolates and the complete genome of multidrug-resistant, heat-resistant strain FAM21845. Appl. Environ. Microbiol..

[8] Zamani H., Salehzadeh A. (2018). Biofilm formation in uropathogenic *Escherichia coli: Association* with adhesion factor genes. Turk. J. Med. Sci..

[9] Candel F.J., Cantón R. (2019). Current approach to Fosfomycin: From bench to bedside. Enferm. Infecc. Microbiol. Clin..

[10] Uruén C., Chopo-Escuín G., Tommassen J., Mainar-Jaime R.C., Arenas J. (2020). Biofilms as promoters of bacterial antibiotic resistance and tolerance. Antibiotics.

[11] Sastry S., Doi Y. (2016). Fosfomycin: Resurgence of an old companion. J. Infect. Chemother..

[12] Candel F.J., David M.M., López J.B. (2019). Nuevas perspectivas para la reevaluación de la fosfomicina: Aplicabilidad en la práctica clínica actual. Rev. Esp. Quimioter..

[13] Iarikov D., Wassel R., Farley J., Nambiar S. (2015). Adverse Events Associated with Fosfomycin Use: Review of the Literature and Analyses of the FDA Adverse Event Reporting System Database. Infect. Dis. Ther..

[14] Amladi A.U., Abirami B., Devi S.M., Sudarsanam T.D., Kandasamy S., Kekre N., Veeraraghavan B., Sahni R.D. (2019). Susceptibility profile, resistance mechanisms & efficacy ratios of Fosfomycin, Nitrofurantoin & Colistin for carbapenem-resistant Enterobacteriaceae causing urinary tract infections. Indian J. Med. Res..

[15] Mezzatesta M.L., La Rosa G., Maugeri G., Zingali T., Caio C., Novelli A., Stefani S. (2017). *In vitro* activity of Fosfomycin Trometamol and other oral antibiotics against multidrug-resistant uropathogens. Int. J. Antimicrob. Agents.

[16] Behzadi P., Urbán E., Gajdács M. (2022). Association between Biofilm-Production and Antibiotic Resistance in Uropathogenic *Escherichia coli* (UPEC): An *In Vitro* Study. Diseases.

[17] Sugathan S., Mandal J. (2019). An *in vitro* experimental study of the effect of Fosfomycin in combination with Amikacin, Ciprofloxacin or Meropenem on biofilm formation by multidrug-resistant urinary isolates of *Escherichia coli*. J. Med. Microbiol..

[18] Gopichand P., Agarwal G., Natarajan M., Mandal J., Deepanjali S., Parameswaran S., Dorairajan L.N. (2019). *In vitro* effect of Fosfomycin on multi-drug resistant Gram-negative bacteria causing urinary tract infections. Infect. Drug. Resist..

[19] Uc-Cachón A.H., Gracida-Osorno C., Luna-Chi I.G., Jiménez-Guillermo J.G., Molina-Salinas G.M. (2019). High prevalence of antimicrobial resistance among Gram-negative isolated bacilli in intensive care units at a tertiary-care hospital in Yucatán Mexico. Medicina.

[20] Sarker S.D., Nahar L., Kumarasamy Y. (2007). Microtitre plate-based antibacterial assay incorporating resazurin as an indicator of cell growth, and its application in the *in vitro* antibacterial screening of phytochemicals. Methods.

[21] Clinical and Laboratory Standards Institute (2017). Performance Standards for Antimicrobial Susceptibility Testing 2017.

[22] Christensen G.D., Simpson W.A., Younger J.J., Baddour L.M., Barrett F.F., Melton D.M., Beachey E.H. (1985). Adherence of coagulase-negative staphylococci to plastic tissue culture plates: A quantitative model for the adherence of staphylococci to medical devices. J. Clin. Microbiol..

[23] Akbari-Ayezloy E., Hosseini-Jazani N., Yousefi S., Habibi N. (2017). Eradication of Methicillin resistant *S. aureus* biofilm by the combined use of Fosfomycin and β-chloro-L-alanine. Iranian. J. Microbiol..

[24] Terlizzi M.E., Gribaudo G., Maffei M.E. (2017). Uropathogenic *Escherichia coli* (UPEC) infections: Virulence factors, bladder responses, antibiotic, and non-antibiotic antimicrobial strategies. Front. Microbiol..

[25] Manseck A.S., Otto W., Schnabel M., Denzinger S., Burger M., Spachmann P.J. (2021). Geriatric patients and symptomatic urinary tract infections: Analysis of bacterial range and resistance rates at a 3rd level of care hospital in Germany. Urol. Int..

[26] Kettani Halabi M., Lahlou F., Diawara I., El Adouzi Y., Marnaoui R., Benmessaoud R., Smyej I. (2021). Antibiotic resistance pattern of extended spectrum beta lactamase producing *Escherichia coli* isolated from patients with urinary tract infection in Morocco. Front. Cell Infect. Microbiol..

[27] Gupta V., Rani H., Singla N., Kaistha N., Chander J. (2013). Determination of extended-spectrum β-lactamases and AmpC production in uropathogenic isolates of *Escherichia coli* and susceptibility to Fosfomycin. J. Lab. Physicians..

[28] Bi W., Li B., Song J., Hong Y., Zhang X., Liu H., Lu H., Zhou T., Cao J. (2017). Antimicrobial susceptibility and mechanisms of Fosfomycin resistance in extended-spectrum β-lactamase-producing *Escherichia coli* strains from urinary tract infections in Wenzhou, China. Int. J. Antimicrob. Agents.

[29] Ballesteros-Monrreal M.G., Arenas-Hernández M.M., Enciso-Martínez Y., Martínez-de la Peña C.F., Rocha-Gracia R., Lozano-Zaraín P., Navarro-Ocaña A., Martínez-Laguna Y., de la Rosa-López R. (2020). Virulence and resistance determinants of uropathogenic *Escherichia coli* strains isolated from pregnant and non-pregnant women from two states in Mexico. Infect. Drug Resist..

[30] Lagunas-Rangel F.A. (2018). Antimicrobial susceptibility profiles of bacteria causing urinary tract infections in Mexico: Single-centre experience with 10 years of results. J. Glob. Antimicrob. Resist..

[31] Díez-Aguilar M., Cantón R. (2019). Nuevos aspectos microbiológicos de la fosfomicina. Rev. Esp. Quimioter..

[32] Horcajada J.P., Montero M., Oliver A., Sorlí L., Luque S., Gómez-Zorrilla S., Grau S. (2019). Epidemiology and treatment of multidrug-resistant and extensively drug-resistant *Pseudomonas aeruginosa* infections. Clin. Microbiol. Rev..

[33] Kengkla K., Kongpakwattana K., Saokaew S., Apisarnthanarak A., Chaiyakunapruk N. (2018). Comparative efficacy and safety of treatment options for MDR and XDR *Acinetobacter baumannii* infections: A systematic review and network meta-analysis. J. Antimicrob. Chemother..

[34] Basak S., Singh P., Rajurkar M. (2016). Multidrug resistant and extensively drug resistant bacteria: A study. J. Pathog..

[35] Blaskovich M.A.T., Pitt M.E., Elliott A.G., Cooper M.A. (2018). Can octapeptin antibiotics combat extensively drug-resistant (XDR) bacteria?. Expert. Rev. Anti. Infec. Ther..

[36] Hall C.W., Mah T.F. (2017). Molecular mechanisms of biofilm-based antibiotic resistance and tolerance in pathogenic bacteria. FEMS Microbiol. Rev..

[37] Tenke P., Köves B., Nagy K., Hultgren S.J., Mendling W., Wullt B., Bjerklund Johansen T.E. (2012). Update on biofilm infections in the urinary tract. World J. Urol..

[38] Naziri Z., Kilegolan J.A., Moezzi M.S., Derakhshandeh A. (2021). Biofilm formation by uropathogenic *Escherichia coli*: A complicating factor for treatment and recurrence of urinary tract infections. J. Hosp. Infect..

[39] Rafaque Z., Abid N., Liaqat N., Afridi P., Siddique S., Masood S., Kanwal S., Dasti J.I. (2020). In-vitro investigation of antibiotics efficacy against uropathogenic *Escherichia coli* biofilms and antibiotic induced biofilm formation at sub-minimum inhibitory concentration of Ciprofloxacin. Infect. Drug. Resist..

[40] Neupane S., Pant N.D., Khatiwada S., Chaudhary R., Banjara M.R. (2016). Correlation between biofilm formation and resistance toward different commonly used antibiotics along with extended spectrum beta lactamase production in uropathogenic *Escherichia coli* isolated from the patients suspected of urinary tract infections visiting Shree Birendra Hospital, Chhauni, Kathmandu, Nepal. Antimicrob. Resist. Infect. Control..

[41] Tewawong N., Kowaboot S., Pimainog Y., Watanagul N., Thongmee T., Poovorawan Y. (2020). Distribution of phylogenetic groups, adhesin genes, biofilm formation, and antimicrobial resistance of uropathogenic *Escherichia coli* isolated from hospitalized patients in Thailand. PeerJ.

[42] Sharma M., Yadav S., Chaudhary U. (2009). Biofilm production in uropathogenic *Escherichia coli*. Indian J. Pathol. Microbiol..

[43] Naves P., del Prado G., Huelves L., Gracia M., Ruiz V., Blanco J., Rodríguez-Cerrato V., Ponte M.C., Soriano F. (2008). Measurement of biofilm formation by clinical isolates of *Escherichia coli* is method-dependent. J. Appl. Microbiol..

[44] Cepas V., López Y., Muñoz E., Rolo D., Ardanuy C., Martí S., Xercavins M., Horcajada J.P., Bosch J., Soto S.M. (2019). Relationship between biofilm formation and antimicrobial resistance in Gram-negative bacteria. Microb. Drug. Resist..

[45] Zhanel G.G., Walkty A.J., Karlowsky J.A. (2016). Fosfomycin: A first-line oral therapy for acute uncomplicated cystitis. Can. J. Infect. Dis. Med. Microbiol..

[46] González M.J., Da Cunda P., Notejane M., Zunino P., Scavone P., Robino L. (2019). Fosfomycin tromethamine activity on biofilm and intracellular bacterial communities produced by uropathogenic *Escherichia coli* isolated from patients with urinary tract infection. Pathog. Dis..

