# Supplementary material for: Efficacy of Fosfomycin against Planktonic and Biofilm-Associated MDR Uropathogenic Escherichia coli Clinical Isolates
Source: Trop Med Infect Dis. 2022 Sep 8;7(9):235. doi: 10.3390/tropicalmed7090235 (PMC9505523; doi:10.3390/tropicalmed7090235)
Supplement: Supplementary file 1 [file tropicalmed-07-00235-s001.zip › tropicalmed-1860208-supplementary.pdf]

Table S1. Resistance profile of MDR UPEC.

ID UPEC	Penicillins	Penicillins + beta-lactamase inhibitors	Antipseudomonal penicillins + beta-lactamase inhibitors	Extended-spectrum cephalosporins; 3rd and 4th generation cephalosporins	Non-extended spectrum cephalosporins; 1st and 2nd generation cephalosporins	Cephamycins	Aminoglycosides	Carbapenems	Fluoroquinolones	Folate pathway inhibitors	Monobactams	Glycylcyclines	Tetracyclines	Phenicols	Phosphonic acids	Polymyxins	Number of drugs to which UPEC is resistant	Number of chemical groups to which UPEC showed resistant at least one antimicrobial	Type of biofilm producer
120	AMP, PIP	SAM	TZP	FEP, CTX, CRO, CAZ	CFX	CTT	AMK, GEN, TOB	IPM, ETP	LVX, CIP	SXT	ATM	TGG	TET				21	13	M
76	AMP, PIP	SAM		FEP, CTX, CRO, CAZ	CFX		GEN, TOB		LVX, CIP	SXT	ATM		TET	CLO	FOF	CST	18	12	M
77	AMP, PIP	SAM	TZP	FEP, CTX, CRO, CAZ	CFX		AMK, TOB		LVX, CIP	SXT	ATM		TET	CLO	FOF		18	12	S
126	AMP, PIP	SAM	TZP	FEP, CTX, CRO, CAZ	CFX		TOB	IPM	LVX, CIP	SXT	ATM		TET	CLO			17	12	S
11	AMP, PIP	SAM		FEP, CTX, CRO, CAZ	CFX	CTT	AMK, TOB, GEN	IPM, MEM, ETP	LVX, CIP	SXT	ATM		TET				20	11	W
87	AMP, PIP	SAM	TZP	FEP, CTX, CRO, CAZ	CFX		AMK, GEN, TOB		LVX, CIP	SXT	ATM		TET	CLO			18	11	M
138	AMP, PIP	SAM	TZP	FEP, CTX, CRO, CAZ	CFX		AMK, GEN, TOB		LVX, CIP	SXT	ATM		TET	CLO			18	11	S
142	AMP, PIP	SAM	TZP	FEP, CTX, CRO, CAZ	CFX	CTT	AMK, GEN, TOB		LVX, CIP	SXT	ATM		TET,				18	11	M
1	AMP, PIP	SAM	TZP	FEP, CTX, CRO, CAZ	CFX		AMK, GEN, TOB		LVX, CIP		ATM		TET		FOF		17	11	W
16	AMP, PIP	SAM	TZP	FEP, CTX, CRO, CAZ	CFX		AMK, TOB		LVX, CIP	SXT	ATM		TET	CLO			17	11	W
109	AMP, PIP	SAM	TZP	FEP, CTX, CRO, CAZ	CFX		AMK, TOB		LVX, CIP	SXT	ATM		TET	CLO			17	11	M
3	AMP, PIP	SAM	TZP	FEP, CTX, CRO, CAZ	CFX			IPM	LVX, CIP	SXT	ATM		TET		FOF		16	11	S
118	AMP, PIP	SAM	TZP	FEP, CTX, CRO, CAZ	CFX	CTT			LVX, CIP	SXT	ATM		TET	CLO			16	11	M
140	AMP, PIP	SAM	TZP	FEP, CTX, CRO, CAZ	CFX	CTT		IPM	LVX, CIP	SXT	ATM		TET				16	11	M
18	AMP, PIP	SAM	TZP	FEP, CTX, CRO, CAZ	CFX		AMK, GEN, TOB		LVX, CIP	SXT	ATM		TET				17	10	W
29	AMP, PIP			FEP, CTX, CRO, CAZ	CFX		GEN, TOB		LVX, CIP	SXT	ATM		TET	CLO	FOF		17	10	W
33	AMP, PIP	SAM	TZP	FEP, CTX, CRO, CAZ	CFX		AMK, GEN, TOB		LVX, CIP	SXT	ATM		TET				17	10	W
61	AMP, PIP	SAM		FEP, CTX, CRO, CAZ	CFX		AMK, GEN, TOB		LVX, CIP	SXT	ATM		TET	CLO			17	10	W
128	AMP, PIP	SAM		FEP, CTX, CRO, CAZ	CFX		AMK, GEN, TOB		LVX, CIP	SXT	ATM		TET	CLO			17	10	M
27	AMP, PIP	SAM	TZP	FEP, CTX, CRO, CAZ	CFX		AMK, TOB		LVX, CIP	SXT	ATM		TET				16	10	W
28	AMP, PIP	SAM	TZP	FEP, CTX, CRO, CAZ	CFX		GEN, TOB		LVX, CIP		ATM		TET	CLO			16	10	M
50	AMP, PIP	SAM		FEP, CTX, CRO, CAZ	CFX		GEN, TOB		LVX, CIP	SXT	ATM		TET	CLO			16	10	M

62	AMP, PIP	SAM	TZP	FEP, CTX, CRO, CAZ	CFX		GEN, TOB		LVX, CIP		ATM		TET	CLO			16	10	M
68	AMP, PIP	SAM	TZP	FEP, CTX, CRO, CAZ	CFX		GEN, TOB		LVX, CIP	SXT	ATM			CLO			16	10	W
72	AMP, PIP	SAM	TZP	FEP, CTX, CRO, CAZ	CFX		GEN, TOB		LVX, CIP	SXT	ATM			CLO			16	10	NP
74	AMP, PIP	SAM		FEP, CTX, CRO, CAZ	CFX		GEN, TOB		LVX, CIP	SXT	ATM		TET	CLO			16	10	M
78	AMP, PIP	SAM	TZP	FEP, CTX, CRO, CAZ	CFX		AMK, TOB		LVX, CIP		ATM		TET	CLO			16	10	NP
93	AMP, PIP	SAM	TZP	FEP, CTX, CRO, CAZ	CFX		GEN, TOB		LVX, CIP	SXT,	ATM		TET				16	10	M
114	AMP, PIP	SAM		FEP, CTX, CRO, CAZ	CFX		GEN, TOB		LVX, CIP	SXT	ATM		TET	CLO			16	10	M
115	AMP, PIP	SAM		FEP, CTX, CRO, CAZ	CFX		GEN, TOB		LVX, CIP	SXT	ATM		TET	CLO			16	10	NP
116	AMP, PIP	SAM	TZP	FEP, CTX, CRO, CAZ	CFX		GEN, TOB		LVX, CIP		ATM		TET	CLO			16	10	W
131	AMP, PIP	SAM	TZP	FEP, CTX, CRO, CAZ	CFX		GEN, TOB		LVX, CIP		ATM		TET		FOF		16	10	W
135	AMP, PIP	SAM	TZP	FEP, CTX, CRO, CAZ	CFX		GEN, TOB		LVX, CIP	SXT	ATM		TET				16	10	NP
32	AMP, PIP		TZP	FEP, CTX, CRO, CAZ	CFX		GEN		LVX, CIP	SXT	ATM		TET	CLO			15	10	NP
34	AMP, PIP	SAM	TZP	FEP, CTX, CRO, CAZ	CFX		TOB		LVX, CIP		ATM		TET	CLO			15	10	W
73	AMP, PIP	SAM	TZP	FEP, CTX, CRO, CAZ	CFX		TOB		LVX, CIP	SXT	ATM		TET				15	10	NP
129	AMP, PIP	SAM	TZP	FEP, CTX, CRO, CAZ	CFX		TOB		LVX, CIP	SXT	ATM		TET				15	10	M
136	AMP, PIP	SAM	TZP	FEP, CTX, CRO, CAZ	CFX				LVX, CIP	SXT	ATM		TET				14	10	S
38	AMP, PIP	SAM	TZP	FEP, CTX, CRO, CAZ	CFX		AMK, GEN, TOB		LVX, CIP		ATM		TET				16	9	W
52	AMP, PIP	SAM		FEP, CTX, CRO, CAZ	CFX		AMK, GEN, TOB		LVX, CIP	SXT	ATM		TET				16	9	W
20	AMP, PIP	SAM	TZP	FEP, CTX, CRO, CAZ	CFX		GEN, TOB		LVX, CIP		ATM		TET				15	9	M
22	AMP, PIP	SAM		FEP, CTX, CRO, CAZ	CFX		GEN, TOB		LVX, CIP		ATM		TET	CLO			15	9	M
37	AMP, PIP	SAM	TZP	FEP, CTX, CRO, CAZ	CFX		AMK, TOB		LVX, CIP		ATM		TET				15	9	NP
48	AMP, PIP	SAM	TZP	FEP, CTX, CRO, CAZ	CFX		GEN, TOB		LVX, CIP		ATM		TET				15	9	W
49	AMP, PIP	SAM		FEP, CTX, CRO, CAZ	CFX		GEN, TOB		LVX, CIP		ATM		TET	CLO			15	9	M
89	AMP, PIP	SAM		FEP, CTX, CRO, CAZ	CFX		GEN, TOB		LVX, CIP	SXT	ATM		TET				15	9	NP
97	AMP, PIP	SAM		FEP, CTX, CRO, CAZ	CFX		GEN, TOB		LVX, CIP	SXT	ATM		TET				15	9	M
117	AMP, PIP	SAM	TZP	FEP, CTX, CRO, CAZ	CFX		GEN, TOB		LVX, CIP		ATM				FOF		15	9	W
133	AMP, PIP	SAM		FEP, CTX, CRO, CAZ	CFX		AMK, TOB		LVX, CIP	SXT	ATM		TET				15	9	NP
139	AMP, PIP	SAM		FEP, CTX, CRO, CAZ	CFX		GEN, TOB		LVX, CIP	SXT	ATM			CLO			15	9	M
7	AMP, PIP			FEP, CTX, CRO, CAZ	CFX				LVX, CIP	SXT	ATM		TET	CLO		CST	14	9	M
19	AMP, PIP	SAM		FEP, CTX, CRO, CAZ	CFX		GEN, TOB			SXT	ATM		TET	CLO			14	9	W
21	AMP, PIP	SAM		FEP, CTX, CRO, CAZ	CFX				LVX, CIP	SXT	ATM		TET	CLO			14	9	M
70	AMP, PIP	SAM		FEP, CTX, CRO, CAZ	CFX				LVX, CIP	SXT	ATM		TET	CLO			14	9	S
54	AMP, PIP	SAM	TZP	FEP, CTX, CRO, CAZ	CFX		GEN, TOB		LVX, CIP				TET	CLO			14	9	W

64	AMP, PIP	SAM		FEP, CTX, CRO, CAZ	CFX				LVX, CIP	SXT	ATM		TET	CLO			14	9	S
66	AMP, PIP	SAM	TZP	FEP, CTX, CRO, CAZ	CFX		TOB		LVX, CIP		ATM		TET				14	9	M
86	AMP, PIP	SAM		FEP, CTX, CRO, CAZ	CFX		TOB		LVX, CIP	SXT	ATM			CLO			14	9	NP
91	AMP, PIP	SAM	TZP	FEP, CTX, CRO, CAZ	CFX		TOB		LVX, CIP	SXT	ATM						14	9	W
95	AMP, PIP	SAM		FEP, CTX, CRO, CAZ	CFX		TOB		LVX, CIP	SXT	ATM			CLO			14	9	W
108	AMP, PIP	SAM	TZP	FEP, CTX, CRO, CAZ	CFX		TOB		LVX, CIP		ATM		TET				14	9	S
112	AMP, PIP	SAM		FEP, CTX, CRO, CAZ	CFX		TOB		LVX, CIP	SXT	ATM		TET				14	9	S
5	AMP, PIP	SAM		FEP, CTX, CRO,CAZ	CFX	CTT			LVX, CIP		ATM		TET	CLO			13	9	W
81	AMP, PIP	SAM	TZP	FEP, CTX, CRO, CAZ	CFX					SXT	ATM		TET	CLO			13	9	M
36	AMP, PIP	SAM	TZP	FEP, CTX, CRO, CAZ	CFX		AMK, GEN, TOB		LVX, CIP		ATM						15	8	M
41	AMP, PIP	SAM	TZP	FEP, CTX, CRO, CAZ	CFX		AMK, GEN		LVX, CIP		ATM						15	8	M
2	AMP, PIP	SAM	TZP	FEP, CTX, CRO, CAZ	CFX		AMK, TOB		LVX, CIP		ATM						14	8	W
26	AMP, PIP	SAM	TZP	FEP, CTX, CRO, CAZ	CFX		GEN, TOB		LVX, CIP		ATM						14	8	W
55	AMP, PIP	SAM		FEP, CTX, CRO, CAZ	CFX		GEN, TOB		LVX, CIP		ATM			CLO			14	8	M
63	AMP, PIP	SAM		FEP, CTX, CRO, CAZ	CFX		GEN, TOB		LVX, CIP		ATM		TET				14	8	M
113	AMP, PIP	SAM		FEP, CTX, CRO, CAZ	CFX		GEN, TOB		LVX, CIP		ATM			CLO			14	8	NP
119	AMP, PIP	SAM	TZP	FEP, CTX, CRO, CAZ	CFX		GEN, TOB		LVX, CIP		ATM						14	8	W
127	AMP, PIP	SAM		FEP, CTX, CRO, CAZ	CFX		GEN, TOB		LVX, CIP		ATM		TET				14	8	W
10	AMP, PIP	SAM		FEP, CTX, CRO, CAZ	CFX				LVX, CIP	SXT	ATM		TET				13	8	M
15	AMP, PIP	SAM		FEP, CTX, CRO, CAZ	CFX				LVX, CIP	SXT	ATM		TET				13	8	W
25	AMP, PIP			FEP, CTX, CRO, CAZ	CFX				LVX, CIP	SXT	ATM		TET	CLO			13	8	NP
43	AMP, PIP	SAM		FEP, CTX, CRO, CAZ	CFX		TOB		LVX, CIP	SXT	ATM						13	8	W
60	AMP, PIP	SAM		FEP, CTX, CRO, CAZ	CFX		TOB		LVX, CIP		ATM		TET				13	8	M
65	AMP, PIP	SAM		FEP, CTX, CRO, CAZ	CFX				LVX, CIP	SXT	ATM		TET				13	8	W
71	AMP, PIP	SAM		FEP, CTX, CRO, CAZ	CFX				LVX, CIP	SXT	ATM		TET				13	8	S
80	AMP, PIP	SAM		FEP, CTX, CRO, CAZ	CFX				LVX, CIP	SXT	ATM		TET				13	8	M
110	AMP, PIP	SAM		FEP, CTX, CRO, CAZ	CFX				LVX, CIP	SXT	ATM		TET				13	8	NP
47	AMP, PIP	SAM	TZP					ERT	LVX, CIP	SXT			TET	CLO			10	8	M
88	AMP, PIP	SAM		FEP, CTX, CRO, CAZ	CFX		GEN, TOB		LVX, CIP		ATM						13	7	W
40	AMP, PIP			FEP, CTX, CRO, CAZ	CFX				LVX, CIP	SXT	ATM		TET				12	7	M
56	AMP, PIP			FEP, CTX, CRO, CAZ	CFX		GEN		LVX, CIP	SXT	ATM						12	7	M
92	AMP, PIP		TZP	FEP, CTX, CRO, CAZ	CFX				LVX, CIP		ATM			CLO			12	7	W
96	AMP, PIP	SAM		FEP, CTX, CRO, CAZ	CFX				LVX, CIP	SXT	ATM						12	7	NP

106	AMP, PIP			FEP, CTX, CRO, CAZ	CFX				LVX, CIP	SXT	ATM		TET				12	7	NP
124	AMP, PIP			FEP, CTX, CRO, CAZ	CFX		GEN		LVX, CIP	SXT	ATM						12	7	M
101	AMP, PIP	SAM		FEP, CTX, CRO, CAZ	CFX					SXT	ATM		TET				11	7	S
105	AMP, PIP	SAM		FEP, CTX, CRO, CAZ	CFX					SXT	ATM		TET				11	7	M
107	AMP, PIP	SAM		FEP, CTX, CRO, CAZ	CFX						ATM		TET	CLO			11	7	M
143	AMP, PIP			FEP, CTX, CRO, CAZ	CFX					SXT	ATM		TET	CLO			11	7	NP
4	AMP, PIP	SAM			CFX		TOB		LVX, CIP	SXT			TET				9	7	NP
39	AMP, PIP			FEP, CTX, CRO, CAZ	CFX				LVX, CIP		ATM		TET				11	6	W
67	AMP, PIP			FEP, CTX, CRO, CAZ	CFX				LVX, CIP		ATM		TET				11	6	M
122	AMP, PIP	SAM					GEN, TOB		LVX, CIP	SXT				CLO			9	6	W
53	AMP, PIP	SAM							LVX, CIP	SXT			TET	CLO			8	6	W
121	AMP, PIP	SAM							LVX, CIP	SXT			TET	CLO			8	6	S

AMP: Ampicillin, PIP: Piperacicllin, SAM: Ampicillin/sulbactam, TZP: Piperacillin/tazobactam, FEP: Cefepime, CTX: Cetofaxime, CRO: Ceftriaxone, CAZ: Ceftazidime, CFX: Cefuroxime, CTT: Cefotetan, AMK: Amikacin, GEN: Gentamicin, TOB: Tobramycin, MEM: Meropenem, IPM: Imipenem, ETP: Ertapenem, LVX: Levofloxacin, CIP: Ciprofloxacin, SXT: Trimethoprim/sulfamethoxazole, ATM: Aztreonam, TGG: Tigecycline, TET: tetracycline, CLO: Chloramphenicol, FOF: Fosfomycin, CST: Colistin. NP: No producer, W: weak, M: moderate, S: strong.
